# Permutation Entropy and Statistical Complexity Analysis of Brazilian Agricultural Commodities

**DOI:** 10.3390/e21121220

**Published:** 2019-12-14

**Authors:** Fernando Henrique Antunes de Araujo, Lucian Bejan, Osvaldo A. Rosso, Tatijana Stosic

**Affiliations:** 1 Departamento de Estatística e Informática, Universidade Federal Rural de Pernambuco, Rua Dom Manoel de Medeiros s/n, Dois Irmãos, Recife, PE 52171-900, Brazil; fhenrique14@gmail.com (F.H.A.d.A.); lucianbb@gmail.com (L.B.); 2 Instituto de Física, Universidade Federal de Alagoas (UFAL). Avenida Lourival Melo Mota s/n, Tabuleiro do Martins, Maceió, AL CEP 57072-900, Brazil; oarosso@gmail.com; 3 Instituto de Medicina Traslacional e Ingeniería Biomedica, Hospital Italiano de Buenos Aires & CONICET. Tte. Gral. Juan Domingo Perón 4190, Ciudad Autónoma de Buenos Aires C1199ABB, Argentina

**Keywords:** permutation entropy, statistical complexity, agricultural commodities, food crisis

## Abstract

Agricultural commodities are considered perhaps the most important commodities, as any abrupt increase in food prices has serious consequences on food security and welfare, especially in developing countries. In this work, we analyze predictability of Brazilian agricultural commodity prices during the period after 2007/2008 food crisis. We use information theory based method Complexity/Entropy causality plane (CECP) that was shown to be successful in the analysis of market efficiency and predictability. By estimating information quantifiers permutation entropy and statistical complexity, we associate to each commodity the position in CECP and compare their efficiency (lack of predictability) using the deviation from a random process. Coffee market shows highest efficiency (lowest predictability) while pork market shows lowest efficiency (highest predictability). By analyzing temporal evolution of commodities in the complexity–entropy causality plane, we observe that during the analyzed period (after 2007/2008 crisis) the efficiency of cotton, rice, and cattle markets increases, the soybeans market shows the decrease in efficiency until 2012, followed by the lower predictability and the increase of efficiency, while most commodities (8 out of total 12) exhibit relatively stable efficiency, indicating increased market integration in post-crisis period.

## 1. Introduction

Agricultural commodity markets have been drawing increased attention over the last decades, both in the scope of mainstream agricultural economics [1,2,3,4] and related fields such as econophysics [5,6,7,8,9,10,11]. The reason for this increased interest is concerns related to the increase of prices of food commodities over the last decades, beginning with the price growth in 2001, followed by the sharp increase during the food crisis of 2007/2008, and subsequently by a new increase in 2010/2011 [12]. The factors that most affected food commodities price spikes over the last decades are increase in biofuel production, which contributed to the food commodity price spike of 2007/2008, the depreciation of U.S. dollar relative to major world currencies, speculations, bad weather in key grain-producing regions, increase in production cost due to high energy prices, and stagnation in productivity growth due to less investment in technology and infrastructure. Finally, trade policies such as export bans on grains by some Asian countries, and import tariffs on non-grain biofuels, such as U.S. tariffs on sugar cane ethanol from Brazil, also contributed to food commodity price spike of 2007/2008 and of 2010/2011 (see e.g., [12] and references therein). Serious consequences of price spikes on food security, especially in developing countries where millions of people were pushed into hunger and poverty [13], often followed by social unrest, political instability, and socio-political conflicts [14], are the major concerns of governments and international organizations [15]. In this sense, every new empirical evidence about agricultural commodities behavior on global and/or local scale provides valuable contribution to worldwide efforts in establishing reliable scientific base to serve as an aid for developing and testing new prediction models that will include different aspects of this extremely complex phenomenon. Our work is designed as a contribution in this direction. Brazil is the world’s third-biggest exporter (after European Union and the United States) of agricultural commodities [16], and it is one of the top producers of agricultural goods including sugar, orange juice, soybeans, coffee, broilers, beef, pork, corn, and cotton [17]. Agricultural markets show a high level of integration, through price transmission and volatility spillover, and Brazil’s agricultural commodities prices were studied within this context. Ceballos et al. [18] analyzed food price volatility transmission (corn, rice, sorghum, and wheat) from the international market to markets of developing countries and found that international price volatility is most likely to be transmitted to markets in South America. Balcombe et al. [19] verified price transmission of wheat, corn, and soybean between the U.S., Argentina, and Brazil during the end of 1980s and beginning of 1990s, generally with causality flowing from the U.S. and Argentina toward Brazil. Agricultural crops have also been used for the production of biofuels. The impact of biofuel programs that were introduced in the United States, Brazil, the European Union, and other countries and the relationship between the food and energy markets have become major topics of economic research [20]. Recently, special attention was given to the relationship between biofuel and its feedstock, such as ethanol and corn in the United States, ethanol and sugar in Brazil, and biodiesel and rapeseed in the European Union [21,22]. Agricultural markets have also been receiving increased attention of researchers from other related fields such as econophysics, providing a new understanding of stochastic processes that govern price dynamics, such as multifractal properties [5,6,7,23], information content [9,10], and network structure [11,24,25]. Previous studies on Brazilian agricultural commodities based on econophysics methods include long-term autocorrelations [8] and long-term cross-correlations between ethanol and sugar [26]. In this work, we analyze predictability of Brazilian agricultural commodity prices during the period after 2007/2008 food crisis. We use information theory based method of Permutation Entropy/Statistical complexity with its representation space called Complexity/Entropy causality plane, which was shown to be successful in the analysis of market efficiency and predictability [10,27,28,29,30].

This paper is organized as follows. In the next section, we present the methodology, then we present data and analysis together with accompanying discussion and, finally, we draw the conclusions.

## 2. Methodology

### 2.1. Permutation Entropy

Permutation entropy (PE) is a method introduced by Bandt and Pompe [31] as Shannon entropy of ordinal patterns of words of a given size (embedding dimension) d, obtained by taking into account the local ordering of consecutive values observed within each word. This method has been widely applied (both in its original form and in its variants) in physiology [32,33], engineering [34,35], geophysics [36], climatology [37,38], hydrology [39], and finances [40,41]. Permutation entropy algorithm proceeds as follows [31].

For a given time series $x_{t}$, t=1,…,T, first T−(d−1) overlapping segments (words) $X_{t}=\left(x_{t},x_{t+1},\ldots ,x_{t+d-1}\right)$ of length d are extracted, and within each segment, the values are sorted in increasing $x_{t+r_{0}}\leq x_{t+r_{1}}\leq \ldots \leq x_{t+r_{d-1}}$, yielding the set of indices $r_{0},\;r_{1},\ldots ,r_{d-1}$. The index sequences $\pi =r_{0},\;r_{1},\ldots ,r_{d-1}$ may assume any of the d! possible permutations of the set {0,1,…,d−1} and are symbolic representatives of the original segments. Relative frequencies of permutations π define the empirical probability distribution p(π), and permutation entropy of order d≥2 is now defined as a Shannon entropy

(1) $$H\left(d\right)=-\underset{\left\{\pi \right\}}{\sum }p\left(\pi \right)\log p\left(\pi \right)$$

where {π} denotes summation over all the d! possible permutations of order d, and logarithm is taken with a base of 2 so that entropy is measured in bits. It follows that H(d) can assume values in the range 0≤H(d)≤logd!, where the lower bound corresponds to strictly increasing or decreasing series (only a single permutation appears), and the upper bound corresponds to a completely random series where all the d! possible permutations have the same probability. The optimal value of embedding dimension d strongly depends on the observed phenomenon, but in order to guarantee good statistics, the typical convention [42] is to use maximum d that satisfies condition T>5d!.

### 2.2. Complexity Entropy Causality Plane

The complexity–entropy causality plane (CECP) was introduced by Rosso et al. [43] as a tool to jointly quantify both information content and structural complexity in a temporal series. It was shown that CECP is useful for distinguishing between stochastic noise and deterministic chaotic behavior [43], leading to many applications in data analysis such as in physiology [44], physics [45,46], oceanography [47], ecology [48], hydrology [49,50,51], and finances [27,28,29,30]. In CECP representation, the horizontal axis is the Permutation entropy, and the vertical axis is a statistical complexity measure, also calculated using Bandt–Pompe probability distribution P. The complexity measure is defined as

(2) $$C\left[P\right]=\frac{J\left[P,U\right]}{J_{max}}H_{s}\left[P\right]$$

where $H_{s}\left[P\right]=H\left[P\right]/\log d!$ is normalized permutation entropy, J[P,U] is the Jensen–Shannon divergence

(3) $$J\left[P,U\right]=\left\{H\left[\frac{\left(P+U\right)}{2}\right]-\frac{H\left[P\right]}{2}-\frac{H\left[U\right]}{2}\right\}$$

which quantifies the distance of the Bandt–Pompe probability distribution P from the uniform distribution U, and $J_{max}$ is the maximum possible value of J[P,U], obtained when one of the components of P is equal to unity, while all the others are equal to zero

(4) $$J_{max}=-\frac{1}{2}\left[\frac{d!+1}{d!}\log\left(d!+1\right)-2\log\left(2d!\right)+\log\left(d!\right)\right]$$

The definition of statistical complexity (2) guarantees that both strictly increasing or decreasing series (for which $H_{s}\left[P\right]=0$) and completely random series (for which J[P,U]=0) have zero complexity. For each given value of the normalized permutation entropy $H_{s}\in \left[0,1\right]$ there is a range of possible values of complexity, $C_{min}\leq C\leq C_{max}$, which gives the lower and upper envelopes in CECP [52]. 

Permutation entropy and structural complexity yield information on two distinct properties of a data set. Permutation entropy quantifies the degree of inherent randomness: more predictable signals that show a tendency to repeat just a few ordinal patterns have lower permutation entropy than less predictable signals that contain many ordinal patterns. For a given permutation entropy value, the statistical complexity quantifies the degree to which there exist privileged ordinal patterns. More precisely, higher complexity for a given permutation entropy value corresponds to larger distance from the uniform distribution, meaning that there are some (privileged) ordinal patterns that appear more often. By calculating these quantities for a given time series, both randomness and the degree of correlational structure in the fluctuations of the system are simultaneously quantified [43]. In the case of financial time series, the localization in CECP provides information about market inefficiency, as an efficient market should be located close to the vertex ($H_{s}\left[P\right]=1,\;C\left[P\right]=0$) that corresponds to a completely random series. The distance from this vertex indicates the degree of market inefficiency (predictability) and was used to compare among stock markets [27] commodities [10] and cryptocurrencies [29]. 

## 3. Data and Analysis

The data used in this work are daily prices of Brazilian agricultural commodities obtained from the Center for Advanced Studies in Applied Economics/Luiz de Queiroz College of Agriculture/University of São Paulo — CEPEA/ESALQ / USP [53]. We analyzed 11 agricultural commodities and also included ethanol, whose price variation is directly related to sugar prices (both commodities are produced from sugarcane) and indirectly (trough relation to energy prices) to other commodities (Table 1). All commodities are analyzed during the same period 01/2010-07/2018, with 2120 data points. 

**Figure 1:**
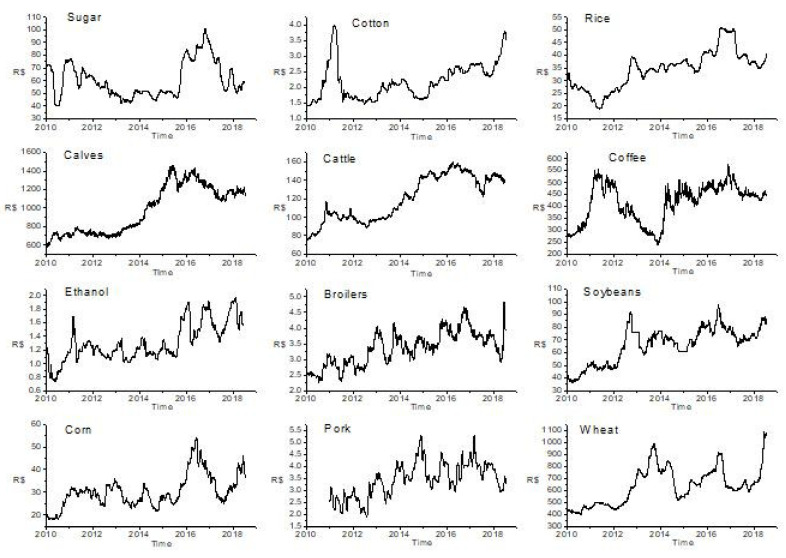
Time series of prices of agricultural commodities recorded daily for the period January 04, 2010, to July 03, 2018.

Following Zunino et al. [10] we analyzed daily commodities prices that are shown in Figure 1. 

**Figure 2:**
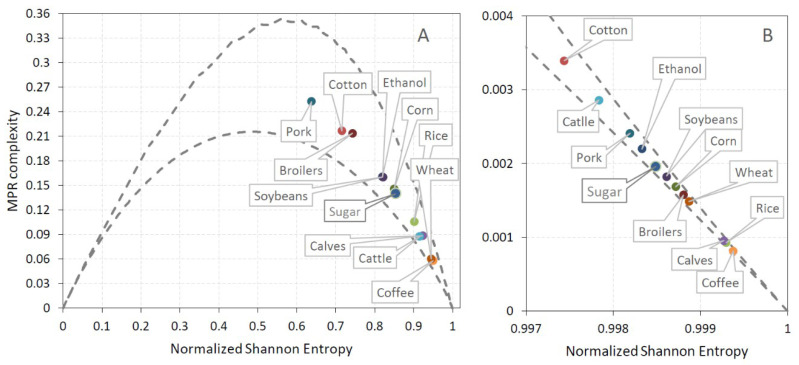
Position in the entropy–complexity plane of (**A**) the original and (**B**) randomized commodities series for embedding dimension d = 4.

**Figure 3:**
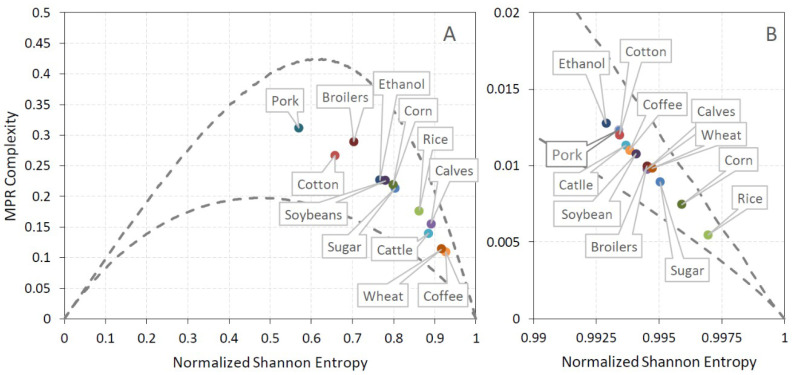
Position in the entropy–complexity plane of (**A**) the original and (**B**) randomized commodities series for embedding dimension d = 5.

Taking into account that each of the analyzed commodity time series contains T = 2120 data points, we chose embedding dimension d = 4 and d = 5 (satisfying the condition T > 5d!) to calculate CECP information quantifiers permutation entropy $H_{s}\left[P\right]$ and statistical complexity C[P]. The locations of the analyzed commodities in the complexity–entropy causality plane for embedding dimension d = 4 and d = 5 together with locations of corresponding randomized series are shown in Figure 2 and Figure 3. Inclusion of points corresponding to randomized series in these figures serves to demonstrate the fact that shuffling moves these points close to the vertex (Hs [P] = 1,C[P] = 0) that corresponds to completely random series (efficient market) and, therefore, the order of values of the original series is far from being random. It can be seen that for both d = 4 (Figure 2) and d = 5 (Figure 3), some specific pairs of commodities ethanol/sugar, soybeans/corn, and cattle/calves have similar positions in CECP. Ethanol and sugar markets are strongly interconnected, as both commodities are produced from the same agricultural crop (sugarcane) and are influenced by global factors (crude oil prices) and specific local features of Brazilian economic development (government policies and technological advances such as flex plants which can easily switch the production from ethanol to sugar, and vice versa) [26,54]. The increase in the price of ethanol (sugar) leads to increased production from sugarcane and, therefore, a lower production of sugar (ethanol), which leads to a long-term imbalance between demand and supply (demand greater than supply) and higher sugar (ethanol) prices. So, it is expected that the two commodities have similar predictability of price variations, which results in their similar position in the CECP plane. In the case of soybeans/corn, one reason for such result could be the fact that in Brazil (which is among the largest producers and consumers of chicken meat) broiler feed is based primarily on corn and soybean meal, which supplies the majority of energy and protein in the diet [55]. Live cattle and calves belong to productivity chain of beef meat and also show similar predictability (position in CECP). Among meat commodities, pork and broilers showed higher predictability (lower entropy) than calf and cattle, while among grains wheat showed lowest predictability (highest entropy). Non-food commodity cotton showed relatively high predictability (low entropy) losing only to pork meat. The Euclidean distance to the CPEP right vertex ($H_{s}=\;$1, C = 0) representing result for completely randomized series can be used as a measure of market inefficiency [10]. The ranking of efficiency of agricultural commodities (ordered by decreasing distance of position in CECP from the right vertex (1,0) that represents an efficient market whose prices follow a random walk) is shown on Table 2, where it is seen that the most efficient (least predictable) is coffee market, and the least efficient (most predictable) is pork meat commodity market. The identical commodity ranking (with exception of cattle and calves) is obtained with different embedding dimensions. 

**Figure 4:**
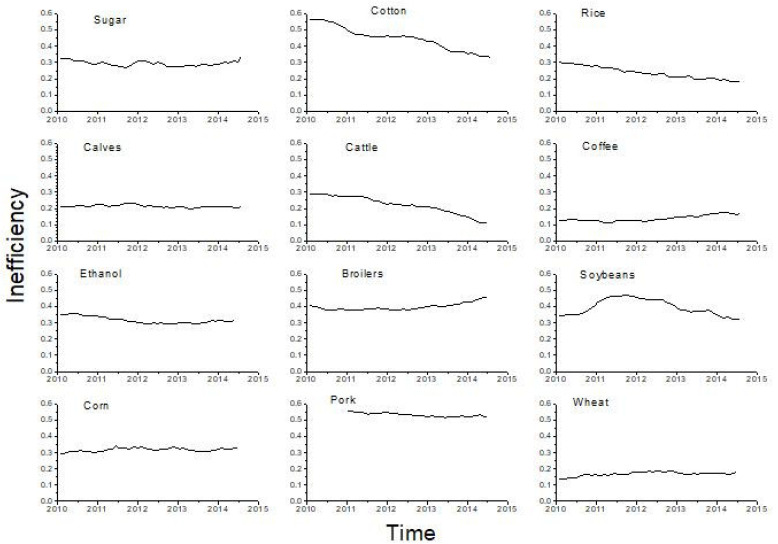
Time evolution of inefficiency measure (distance from complexity–entropy causality plane (CECP) point $H_{s}=\;$1, C = 0) for commodities series for embedding dimension d = 5. The points on the graph correspond to the beginning of the corresponding 1000 data point windows.

In order to see how the efficiency of commodities changes over time, we applied CECP analysis in sliding windows of size of 1000 data (around four business years) width a step of 20 data (around one business month), and in each window we calculated the distance of CECP position from the right vertex (1,0). We chose the window size of four business years with a step of one business month in order to be able to compare our results with those from previous studies [10] and to provide sufficiently long time series for permutation entropy calculations. The time evolution of this distance (inefficiency measure) is shown on Figure 4 from which we can observe that 8 out of 12 markets (broilers, pork, ethanol, corn, sugar, calves, wheat, and coffee) exhibit relatively stable inefficiency during the analyzed period, with pork showing the highest inefficiency and wheat and coffee the lowest inefficiency. The inefficiency of cotton, rice, and cattle market decreases, but overall cattle and rice markets are less inefficient than cotton market (indicated by lower values of the inefficiency index). The soybeans market shows the increase in inefficiency (higher predictability) until 2012, followed by the decrease of inefficiency (lower predictability) for the rest of the studied period. This pattern coincides with the variation of soybeans prices that were lower and more predictable during the period 2010–2012, followed by the period of higher prices and lower market inefficiency.

## 4. Conclusions and Discussion

During the last decade, commodities became included in portfolio diversification. Commodity financialization (increase in investments in commodities through financial instruments), which took effect between 2004 and 2005, has generated an increase in integration within commodity markets and, in particular, in the agricultural commodity sector [56]. Differently than other types of commodities, agricultural commodities exhibited unexpected extreme fluctuations, especially during the period 2007–2009, which makes market participants such as producers, consumers, and investors to be seriously concerned about the movements of agricultural commodities as well as their co-movements on both single market level and among different markets. In this work, we investigate price variations in the Brazilian agricultural market, specifically predictability of Brazilian agricultural commodities for the period after the 2007/2008 food crisis. We use the complexity–entropy causality plane (CECP) method, which is a model-free tool to jointly quantify information content and structural complexity in temporal series. The main results of this work are: (i) specific pairs of commodities ethanol/sugar, soybeans/corn, and cattle/calves have similar positions in CECP, reflecting their interconnection within Brazilian agricultural market; (ii) comparing the deviation from the right end of CECP (that corresponds to a completely random process) reveals that the most efficient (least predictable) is the coffee market and the least efficient (most predictable) is the pork meat market; (iii) by analyzing temporal evolution of commodity prices in the complexity–entropy causality plane, we observed that during the post-crisis period the efficiency of cotton, rice, and cattle market increases, the soy market shows the decrease in efficiency (higher predictability) until 2012, followed by the increase of efficiency (lower predictability), while other commodities exhibit relatively stable efficiency (with pork market showing the lowest efficiency, and wheat and coffee markets the highest efficiency). Zunino et al. [10] analyzed predictability of international commodity market including several agricultural commodities, which comparing with our results on Brazilian agricultural commodities showed higher market efficiency. However, the analyzed period in Reference [10] was 1991–2009, before and during 2007/2008 food crisis and our results from 2010–2018 data reveal the post-crisis market efficiency evolution. The decrease in agricultural market efficiency after the crisis was also reported in other studies. Ceballos et al. [18] analyzed food price volatility transmission (corn, rice, sorghum, and wheat) from international market to markets of developing countries and found that international price volatility is most likely to be transmitted to markets in South America. They also found that except for sorghum, which showed only a moderate increase, volatility for the rest of the commodities increased by more than 30% after the crisis, indicating lower market efficiency. 

Comparing our results with those of Zunino et al. [10], we observed that agricultural markets become less efficient (more predictable) after 2007/2008 food crisis, which is in agreement with the results of some recent studies [18]. Although the importance of understanding price variations of agricultural commodities and its contributing factors were widely recognized, yielding a large number of results in agricultural economics, in econophysics literature, most studies concentrate on behavior of stock market indices and prices of individual stocks, while commodities markets are much less explored. Our results contribute to a better understanding of agricultural commodities as complex systems, by providing the information (extracted from CECP) about both randomness and the degree of correlational structure in the price fluctuations. For the particular case of the Brazilian market, we identify intervals of increasing and decreasing efficiency (lower and higher predictability) in the commodity dynamics during the post-crisis period. The information extracted from CECP reveals that some commodities behave in a similar way (exhibit similar values of information quantifiers), which may be valuable for investors and policymakers for investigating anomalous market movements such as bubbles or speculations. Future studies should focus on differences and similarities with agricultural markets in other countries that have strong trade with Brazil, as well as the influence of other financial variables from domestic and international markets. 

## Figures and Tables

**Table 1:** List of agricultural commodities with a price in Brazilian reals (R$) per unit of measurement.

Name	Currency (Brazilian Real–R$)/ Unit of Measure
Sugar	R$/bag of 50 kg
Cotton	R$/pound (0.453597 kg)
Rice	R$/bag of 50 kg
Calves	R$/head
Cattle	R$/15 kg
Coffee	R$/bag of 60 kg
Ethanol	R$/liter
Broilers	R$/Kg
Corn	R$/bag of 60 kg
Soybeans	R$/bag of 60 kg
Pork	R$/Kg
Wheat	R$/Ton

**Table 2:** Commodities ranking by decreasing efficiency. Values of permutation entropy $H_{s}$, and statistical complexity C and distance from vertex (1,0) are calculated for d = 4 and d = 5.

	*d* = 4				*d* = 5			
Position	Commodities	PE	CP	Dist. to (1,0)	Commodities	PE	CP	Dist. to (1,0)
1.	Coffee	0.950	0.058	0.076	Coffee	0.926	0.109	0.132
2.	Wheat	0.946	0.060	0.081	Wheat	0.916	0.114	0.142
3.	Calves	0.923	0.088	0.117	Cattle	0.884	0.139	0.181
4.	Cattle	0.915	0.088	0.122	Calves	0.891	0.155	0.189
5.	Rice	0.901	0.106	0.144	Rice	0.862	0.176	0.224
6.	Sugar	0.854	0.140	0.202	Sugar	0.803	0.213	0.290
7.	Corn	0.850	0.146	0.209	Corn	0.798	0.219	0.298
8.	Soybeans	0.822	0.160	0.240	Soybeans	0.780	0.226	0.316
9.	Ethanol	0.820	0.161	0.241	Ethanol	0.766	0.227	0.326
10.	Broilers	0.744	0.214	0.334	Broilers	0.703	0.289	0.414
11.	Cotton	0.716	0.217	0.357	Cotton	0.657	0.267	0.434
12.	Pork	0.638	0.253	0.441	Pork	0.570	0.312	0.531
